# White blood cell and cell-free DNA analyses for detection of residual disease in gastric cancer

**DOI:** 10.1038/s41467-020-14310-3

**Published:** 2020-01-27

**Authors:** Alessandro Leal, Nicole C. T. van Grieken, Doreen N. Palsgrove, Jillian Phallen, Jamie E. Medina, Carolyn Hruban, Mark A. M. Broeckaert, Valsamo Anagnostou, Vilmos Adleff, Daniel C. Bruhm, Jenna V. Canzoniero, Jacob Fiksel, Marianne Nordsmark, Fabienne A. R. M. Warmerdam, Henk M. W. Verheul, Dick Johan van Spronsen, Laurens V. Beerepoot, Maud M. Geenen, Johanneke E. A. Portielje, Edwin P. M. Jansen, Johanna van Sandick, Elma Meershoek-Klein Kranenbarg, Hanneke W. M. van Laarhoven, Donald L. van der Peet, Cornelis J. H. van de Velde, Marcel Verheij, Remond Fijneman, Robert B. Scharpf, Gerrit A. Meijer, Annemieke Cats, Victor E. Velculescu

**Affiliations:** 1 The Sidney Kimmel Comprehensive Cancer Center, Johns Hopkins University School of Medicine, Baltimore, MD 21287 USA; 2 Department of Pathology, Cancer Center Amsterdam, Amsterdam UMC, Vrije Universiteit, Amsterdam, Netherlands; 3 Department of Medicine, Johns Hopkins University School of Medicine, Baltimore, MD 21287 USA; 4 Department of Oncology, Aarhus University Hospital, Aarhus, Denmark; 5 Department of Medical Oncology, Zuyderland Medical Centre, Sittard-Geleen/Heerlen, Netherlands; 6 Department of Medical Oncology, Cancer Center Amsterdam, Amsterdam UMC, Vrije Universiteit, Amsterdam, Netherlands; 7 Department of Hematology, Radboud University Nijmegen Medical Centre, Nijmegen, Netherlands; 8 Department of Internal Medicine, St. Elisabeth-Tweesteden Ziekenhuis, Tilburg, Netherlands; 9 Department of Internal Medicine, Onze Lieve Vrouwe Gasthuis, Amsterdam, Netherlands; 10 Department of Internal Medicine, HAGA hospital, The Hague, Netherlands; 11 Department of Radiation Oncology, Netherlands Cancer Institute, Amsterdam, Netherlands; 12 Department of Surgery, Netherlands Cancer Institute, Amsterdam, Netherlands; 13 Department of Surgery, Leiden University Medical Center, Leiden, Netherlands; 14 Department of Medical Oncology, Cancer Center Amsterdam, Amsterdam UMC, Amsterdam, Netherlands; 15 Department of Surgery, Cancer Center Amsterdam, Amsterdam UMC, Vrije Universiteit, Amsterdam, Netherlands; 16 Department of Pathology, Diagnostic Oncology, Netherlands Cancer Institute, Amsterdam, Netherlands; 17 Department of Gastrointestinal Oncology, Netherlands Cancer Institute, Amsterdam, Netherlands; 18 Present Address: Department of Internal Medicine, Leiden University Medical Center, Leiden, Netherlands

**Keywords:** Prognostic markers, Gastric cancer

## Abstract

Liquid biopsies are providing new opportunities for detection of residual disease in cell-free DNA (cfDNA) after surgery but may be confounded through identification of alterations arising from clonal hematopoiesis. Here, we identify circulating tumor-derived DNA (ctDNA) alterations through ultrasensitive targeted sequencing analyses of matched cfDNA and white blood cells from the same patient. We apply this approach to analyze samples from patients in the CRITICS trial, a phase III randomized controlled study of perioperative treatment in patients with operable gastric cancer. After filtering alterations from matched white blood cells, the presence of ctDNA predicts recurrence when analyzed within nine weeks after preoperative treatment and after surgery in patients eligible for multimodal treatment. These analyses provide a facile method for distinguishing ctDNA from other cfDNA alterations and highlight the utility of ctDNA as a predictive biomarker of patient outcome to perioperative cancer therapy and surgical resection in patients with gastric cancer.

## Abstract (web summary)

Identifying patients that will respond to a particular therapy remains a key challenge in precision oncology. Here, in gastric cancer, the authors show that circulating tumour DNA can predict recurrence, provided that the signal from white blood cells is filtered out.

## Introduction

A major challenge after multimodal curative treatment for resectable gastric cancer is identifying patients with microscopic residual disease at high risk of recurrence after surgery^1–4^. Currently available imaging techniques and traditional blood biomarkers to capture minimal residual disease (MRD) state after surgery have poor sensitivity and do not play a role in clinical practice^5^. Histopathological assessment of the effects of neoadjuvant chemotherapy on resection specimens has become an important tool to provide prognostic information^6–8^. However, microscopic residual tumor, lymph node infiltration, and poor histopathological response do not measure the real-time presence of residual disease. More recent approaches such as detection of circulating tumor-derived DNA (ctDNA) through liquid biopsies may provide new opportunities for identifying patients that would benefit from adjuvant treatment options and further follow-up^9–12^.

Theoretically, the ability to non-invasively detect tumor-specific alterations in the circulation after neoadjuvant chemotherapy and surgery has the potential to rapidly and dynamically inform the presence of MRD or preclinical metastases^13,14^. Cell-free ctDNA is released from tumor cells into the circulation and has been detected in patients with early- and late-stage cancers^15–19^. A key challenge of liquid biopsy approaches has been developing methods to detect and characterize small amounts of ctDNA in large populations of cell-free DNA (cfDNA). A variety of studies have focused on changes in ctDNA during the course of therapy, but mostly in the setting of metastatic disease and largely centered on the analysis of a limited number of genomic positions that may only represent a subset of clones of the tumor^20–28^. More recent studies have used panels of commonly mutated driver genes to allow detection of multiple driver clones, typically at the time of diagnosis, after surgery, or at disease progression^16,18,27,29–34^. However, blood-based deep sequencing approaches have raised concerns about detection and misclassification of white blood cell (WBC)-derived variants in cfDNA associated with clonal hematopoiesis of indeterminate potential^18,35–37^. Such studies have typically assessed WBC DNA and cfDNA in patients with cancer at a single timepoint^18,38–40^ or in healthy individuals^41^, and did not attempt to evaluate these during the course of therapy to predict clinical outcome.

In the present study, we apply a matched cfDNA and WBC sequencing approach to accurately detect ctDNA alterations after preoperative chemotherapy and after surgery in patients with resectable gastric cancer. We hypothesize that ctDNA detection after completion of preoperative treatment as well as MRD detection after surgery can predict recurrence and survival in patients with resectable gastric cancer treated with multimodal therapeutic regimens. Overall, these analyses evaluate a strategy to distinguish ctDNA alterations from cfDNA variants related to clonal hematopoiesis and investigate whether ctDNA elimination before or after surgery can serve as a predictive biomarker of patient outcome to perioperative treatment.

## Results

### Overall approach

The current study was an exploratory analysis of the predictive value of ctDNA assessment in a subset of patients from the CRITICS (ChemoRadiotherapy after Induction chemoTherapy In Cancer of the Stomach) study (NCT00407186), an investigator-initiated, open-label, multi-center, phase III randomized controlled trial of perioperative chemotherapy (chemotherapy group) vs. preoperative chemotherapy with postoperative chemoradiotherapy (chemoradiotherapy group) for patients with resectable gastric cancer^42^. Between 11 January 2007 and 17 April 2015, a total of 788 patients from 56 hospitals in the Netherlands, Sweden, and Denmark were randomized upfront to receive three preoperative 21-day cycles of intravenous epirubicin, cisplatin or oxaliplatin, and oral capecitabine followed by three postoperative cycles of intravenous epirubicin, cisplatin or oxaliplatin, and oral capecitabine (chemotherapy group) or to receive the same preoperative regimen followed by radiation combined with daily capecitabine and weekly cisplatin (chemoradiotherapy group).

**Fig. 1:**
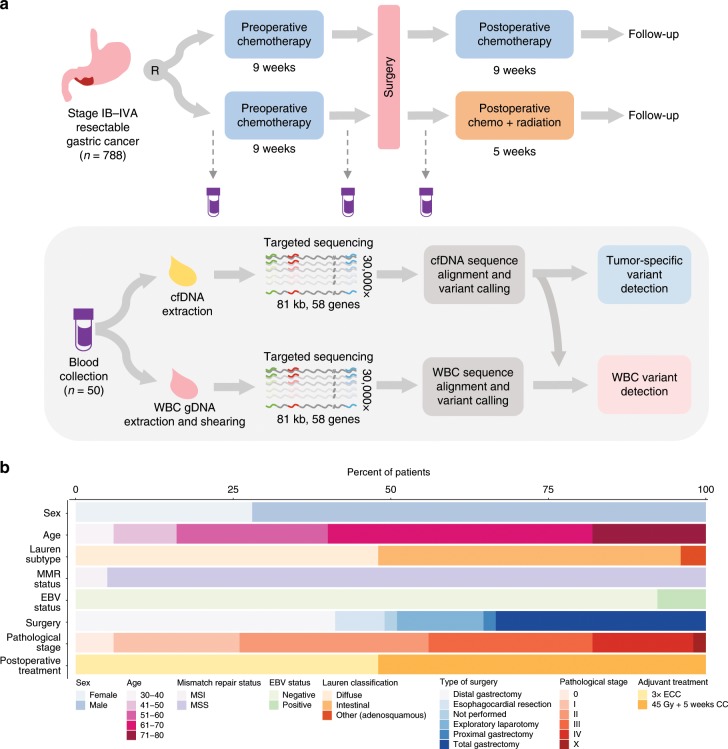
Analysis of cfDNA in patients with resectable gastric cancer. Study schematic (**a**). Patients with confirmed stage IB–IVA gastric adenocarcinoma eligible for perioperative treatment with systemic chemotherapy were randomized upfront to receive three cycles of preoperative chemotherapy, followed by three cycles of postoperative chemotherapy, or to receive the same preoperative regimen, followed by postoperative radiotherapy combined with chemotherapy. A blood draw was collected for each patient at the time of study enrollment (baseline), after three cycles of preoperative chemotherapy (preoperative timepoint), and after surgery (postoperative timepoint). Blood samples were initially processed to allow extraction of cfDNA from plasma and genomic DNA (gDNA) from white blood cells (WBC). Both cfDNA and WBC gDNA libraries were captured with custom RNA oligo pools encompassing 80,930 bases across 58 cancer driver genes. Capture libraries were sequenced at high coverage (>30,000×), followed by sequence alignment, error correction, and variant calling. Mutations detected in WBCs were identified in cfDNA and removed, allowing the identification of tumor-specific mutations in cfDNA. Clinicopathological characteristics, type of surgical treatment, pathological stage, and type of postoperative treatment for the subset of patients were analyzed in this translational study (*n* = 50) (**b**). MSI, microsatellite instability; MSS, microsatellite stability; EBV, Epstein–Barr virus; ECC, epirubicin, cisplatin, capecitabine; CC, cisplatin, capecitabine.

As a proof-of-principle study, we sequenced and analyzed matched cfDNA and WBC samples from 50 treatment-naive patients from the Netherlands who fulfilled the following criteria: (i) were enrolled in one of the two arms of the study and received three cycles of preoperative chemotherapy; (ii) had plasma available from at least two consecutive timepoints processed within 24 h after blood collection for cfDNA analyses; (iii) had buffy coat or whole blood at baseline for WBC analysis; and (iv) had clinical follow-up longer than 18 months if they were free of disease after therapy (Fig. 1a; Supplementary Fig. 1; Supplementary Data 1). Our goal was to predict survival outcomes based on ctDNA assessment after preoperative therapy and MRD analyses after surgery with curative intent. Of the patients analyzed, 25 had diffuse subtype, 24 had intestinal subtype according to Lauren’s classification, and one was diagnosed with adenosquamous gastric carcinoma (Fig. 1b; Supplementary Data 1). Twenty-one patients were surgically treated with distal gastrectomy, 17 with total gastrectomy, four with esophagocardiac resection, and one with proximal gastrectomy. Despite initial eligibility at the time of treatment enrollment, six patients showed evidence of advanced disease during the exploratory laparotomy and were not submitted to surgical resection. One additional patient did not undergo surgical treatment for unknown reasons. Histopathological regression after preoperative therapy was determined according to Mandard’s tumor regression grade (TRG). Three patients achieved complete regression after three cycles of preoperative chemotherapy at the time of surgery (TRG 1), while 10, 15, 13, and 2 patients presented with pathological stage I, II, III, and IV, respectively. Centrally reviewed pathological assessment of resection specimens after three cycles of preoperative chemotherapy showed that 20 patients did not have evidence of lymph node involvement (ypN0), while 23 patients had lymph node infiltration, including 10 patients with ypN1 (including 3 ypN1mi), 7 patients with ypN2, and 6 patients with ypN3 disease. Twenty-six patients received postoperative treatment with radiation combined with cisplatin and capecitabine, and 24 patients were postoperatively treated with three cycles of epirubicin, cisplatin, and capecitabine without radiation after surgery.

For each patient, plasma and buffy coat were collected at the time of trial enrollment (baseline timepoint), after patients received three cycles of preoperative chemotherapy (preoperative timepoint), and after surgery but before the initiation of the adjuvant treatment (postoperative timepoint) (Fig. 1a; Supplementary Data 1 and 2). We developed an approach to identify tumor-specific alterations in the circulation independent of tissue analyses by parallel deep sequencing of cfDNA and WBCs, followed by identification of cfDNA alterations and removal of hematopoietic-related changes detected in WBCs. For sequencing analyses of cfDNA and WBCs, we used a next-generation targeted error correction sequencing approach to evaluate 58 cancer driver genes (Fig. 1a; Supplementary Data 3–5)^18^. This method is based on targeted capture and deep sequencing (>30,000×) of DNA fragments to identify single base substitutions and small insertions or deletions in cfDNA across 80,930 bp of coding gene regions while distinguishing these from PCR amplification and sequencing artifacts^18^. For alterations detected in cfDNA that were not identified by matched WBC sequencing, we determined the probability that such an alteration was tumor derived from a Bayesian statistical model using the frequency of altered alleles and total coverage of cfDNA and WBC sequences (see Methods).

To estimate the theoretical sensitivity of detection of the sequencing approach in gastric cancer, we determined the proportion of gastric adenocarcinomas in the TCGA Pan-Cancer Atlas^43^ with alterations in one or more of the 58 analyzed genes. These analyses showed that our targeted panel would have a sensitivity of ~88% as 384 of 436 gastric cancer cases had at least one alteration in these genes (Supplementary Fig. 2). Overall, we observed that median levels of mutant allele fractions at baseline were significantly higher in patients with intestinal subtype when compared to diffuse subtype (0.295 vs. 0%, *p* = 0.015, Wilcoxon’s rank-sum test) (Supplementary Fig. 3a). There was no statistically significant difference among levels of mutant allele fractions among well, moderately, or poorly differentiated tumors (*p* = 0.07, Kruskal–Wallis test) (Supplementary Fig. 3b). In our study, patients with intestinal and diffuse gastric adenocarcinoma experienced similar event-free (Supplementary Data 1; Supplementary Fig. 3c) and overall survival (Supplementary Data 1; Supplementary Fig. 3d). Consistent with the findings of the original trial^42^, we did not observe significant differences in survival outcomes related to the postoperative treatment arm in which patients had been randomized (Supplementary Data 1; Supplementary Fig. 3e, f).

### Detection of clonal hematopoiesis and tumor-specific alterations

**Fig. 2:**
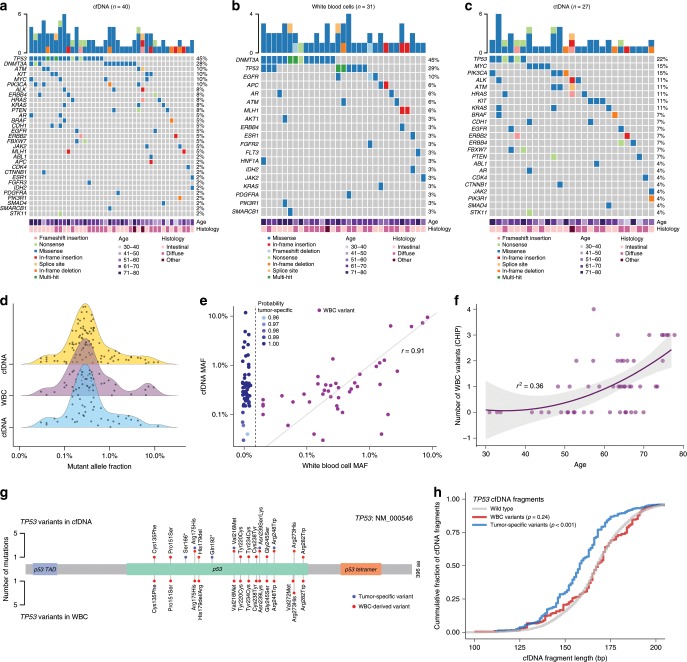
Identification of white blood cell and ctDNA variants in cfDNA. Ultrasensitive targeted sequencing was used to detect mutations in cfDNA (**a**) and WBCs (**b**) in 50 patients, with only those cases having alterations indicated. Tumor-specific mutations in ctDNA were identified in 27 individuals after subtraction of WBC-derived variants in cfDNA (**c**). Density plots showing the mutant allele fraction distribution of cfDNA variants (top, yellow), WBC variants (middle, purple), and resulting ctDNA variants (bottom, blue) (**d**). Levels of mutant allele fractions in WBCs (horizontal axis) and their correspondent levels in cfDNA (vertical axis) suggest that WBC alterations are identified at similar levels in cfDNA (Pearson‘s correlation coefficient = 0.91, *p* < 0.001). The probability that an identified variant is tumor-derived when the alteration is not detected in WBCs is indicated by the shading of the blue dots (**e**). Association between age (horizontal axis) and absolute number of WBC variants detected in each patient (vertical axis) suggests that the number of WBC alterations increase with age (*r*^2^ *=* 0.36, exponential correlation) (**f**). Positions and frequencies of mutations in *TP53* detected in cfDNA (top plot) and WBCs (bottom plot) demonstrate that the majority of *TP53* alterations in cfDNA are from WBCs. One *TP53* splice site mutation detected in both datasets is not shown (**g**). Cumulative fraction of cfDNA fragments based on cfDNA fragment length (bp) shows an altered distribution for cfDNA fragments harboring tumor-derived *TP53* alterations (blue) compared to WBC *TP53* variants (red) and wild-type *TP53* sequences (*p* < 0.001, Kolmogorov–Smirnov test) (**h**).

We evaluated cfDNA in all 50 patients at baseline and after three cycles of preoperative chemotherapy. At baseline, we detected sequence alterations in cfDNA from 40 patients (80%) (Fig. 2a; Supplementary Data 6) and in WBCs from 31 patients (62%) (Fig. 2b; Supplementary Data 7). After removing WBC-derived alterations from cfDNA data (Supplementary Data 8), we detected 54 alterations that were likely tumor-specific in 27 patients (54%) (Fig. 2c; Supplementary Data 9). The most frequently altered genes detected in ctDNA were *TP53* (22%), *MYC* (15%), *PIK3CA* (15%), *KRAS* (11%), *HRAS* (11%), *BRAF* (11%), *ALK* (11%), *ATM* (11%), *KIT* (11%), and *CDH1* (7%) (Fig. 2c; Supplementary Data 9). In accord with the molecular classification of gastric adenocarcinomas proposed by the TCGA^44^, we found a higher frequency (60%) of *PIK3CA* mutations in ctDNA of patients with Epstein–Barr virus (EBV)-positive (*n* = 3) or MSI-high tumors (*n* = 2) compared to the frequency (3%) in EBV-negative (*n* = 36) and MSS tumors (*n* = 37).

Genes most frequently affected in WBCs were *DNMT3A* (45%), *TP53* (29%), *EGFR* (10%), *APC* (6%), *AR* (6%), *ATM* (6%), and *MLH1* (6%) (Fig. 2b; Supplementary Data 7). Functional prediction analyses of these alterations using multiple approaches revealed that the majority of changes were likely to be deleterious or pathogenic^45,46^ (Supplementary Fig. 4a and Supplementary Data 10). The median mutant allele fraction among 43 WBC-derived variants was 0.31% (interquartile range (IQR) 0.18–0.63%), which was similar to the median mutant allele fraction among 53 tumor-specific variants identified in cfDNA (0.31%, IQR 0.20–0.55%, *p* = 0.96, Wilcoxon’s rank-sum test) (Fig. 2d). We observed a high correlation between levels of mutant allele fractions in WBCs and levels of corresponding alterations in cfDNA (Pearson’s correlation coefficient = 0.91) (Fig. 2e). As expected, the number of alterations detected in WBCs increased with age (*r*^2^ = 0.36, exponential correlation) (Fig. 2f). Interestingly, we detected WBC variants in *DNMT3A*, *TP53*, *ERBB4*, *MLH1*, *PDGFRA*, *FGFR3*, *ESR1*, *IDH2*, and *ATM* among multiple timepoints analyzed in 11 patients that did not harbor any tumor-specific alterations in cfDNA (Supplementary Fig. 4b–l).

We detected 21 sequence alterations in *TP53* in cfDNA, including 17 missense mutations, two nonsense mutations, one in-frame deletion, and one splice site mutation (Fig. 2g; Supplementary Data 6). Of the cfDNA sequence changes observed in *TP53*, we identified 15 in WBC sequences as well as three alterations in WBC’s that were not present in matched cfDNA (Fig. 2g; Supplementary Data 7). From the 21 *TP53* alterations initially detected in cfDNA at baseline, only six were identified as tumor-specific mutations, including two with stop alterations (Q192* and S166*) as well as four missense mutations (R175H, V216M, N239S, R248W). We further evaluated fragment length distributions of the 21 *TP53* alterations detected in cfDNA. We observed that fragments harboring tumor-specific *TP53* mutations in the circulation were significantly shorter than fragments harboring *TP53* variants associated with clonal hematopoiesis (*p* < 0.001, Kolmogorov–Smirnov test), as well as fragments harboring wild-type *TP53* coding regions (*p* < 0.001, Kolmogorov–Smirnov test) (Fig. 2h; Supplementary Data 7–9). Comparison of all ctDNA and WBC variants in this study to the COSMIC database of somatic alterations revealed an enrichment of solid tumors having alterations at the same positions as ctDNA variants, while hematological malignancies were enriched with alterations at positions of WBC changes (*p* < 0.0001, *χ*^2^ test) (Supplementary Fig. 5a). Overall, detection of WBC variants and tumor-derived ctDNA variants at baseline did not reveal statistically significant differences in event-free or overall survival (Supplementary Fig. 5b–e).

### Preoperative ctDNA is a biomarker for pathological response

**Fig. 3:**
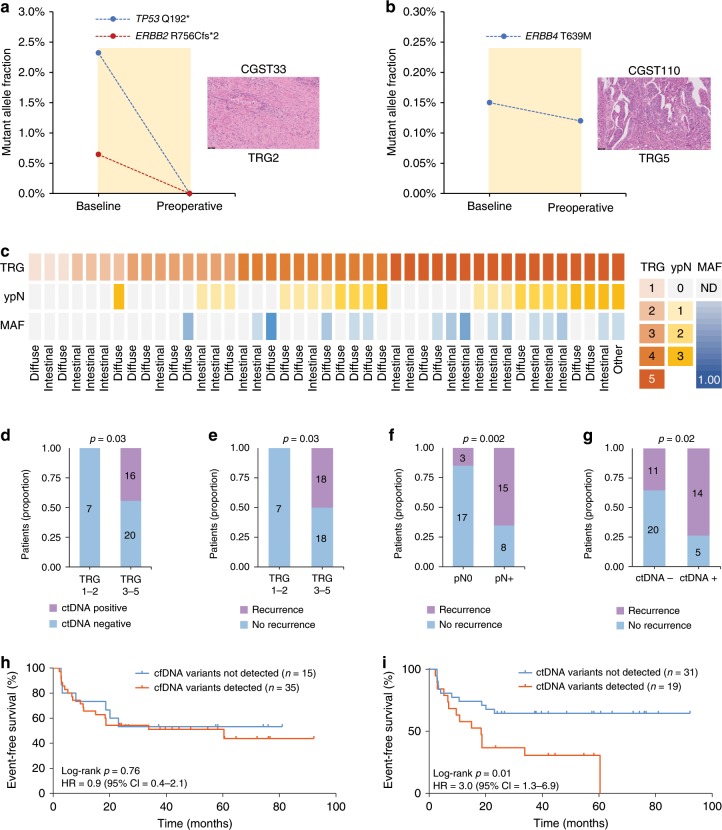
Preoperative ctDNA as a biomarker for pathologic response and clinical outcome in gastric cancer. Levels of ctDNA variants at baseline and the preoperative timepoint in a molecular responder (CGST33) (**a**) and in a non-responder (CGST110) (**b**). Variant MAFs in the molecular responder show elimination of ctDNA, while ctDNA levels are relatively unchanged in the molecular non-responder. A representative H&E image (×20 magnification) depicting Mandard’s tumor regression grade is shown for each case on the right. Heatmap representing the pathological features (TRG and lymph node status) and highest mutant allele fraction detected for each of the 43 patients who underwent surgical resection. Lauren’s classifications are depicted in the bottom for each case (**c**). Dichotomized association between degree of tumor regression and ctDNA status at the preoperative timepoint (*p* = 0.03, Fisher’s exact test) (**d**), between degree of tumor regression and disease recurrence (*p* = 0.03, Fisher’s exact test) (**e**), between pathological lymph node status and disease recurrence (*p* *=* 0.002, Fisher’s exact test) (**f**), and between ctDNA status at the preoperative timepoint and disease recurrence (*p* = 0.02, Fisher’s exact test) (**g**). Kaplan–Meier estimates for event-free survival of patients with detected vs. not detected variants at the preoperative timepoint using all cfDNA sequence changes (log-rank *p* = 0.76; HR = 0.9; 95% CI = 0.4–2.1) (**h**) or using only ctDNA alterations identified from the WBC-filtered approach (log-rank *p* *=* 0.01; HR = 3.0; 95% CI = 1.3–6.9) (**i**).

After identification of ctDNA alterations using the parallel sequencing of cfDNA and WBCs indicated above, we evaluated ctDNA levels before and after preoperative chemotherapy. Of the 30 patients with measurable ctDNA at baseline or at the preoperative timepoint after filtering WBC sequence alterations (Fig. 2c), 11 experienced a complete elimination of ctDNA levels after 9 weeks of systemic treatment (Supplementary Figs. 6 and 7; Supplementary Data 5). As an example, patient CGST33, who presented with intestinal subtype gastric adenocarcinoma at diagnosis, had mutant allele fraction concentrations of 2.32% and 0.64% for *TP53* Q192* and *ERBB2* R756Cfs*2, respectively, that were completely eliminated at the preoperative timepoint. This drop in ctDNA occurred in conjunction with a major pathological response (TRG 2) in the specimen obtained at the time of surgery (Fig. 3a). In contrast, 19 patients had detectable ctDNA at the preoperative timepoint (Supplementary Figs. 6 and 7; Supplementary Data 5), including as an example in patient CGST110 who had mutant allele fractions of 0.15% for *ERBB4* T639M at baseline and 0.12% at the preoperative timepoint (Fig. 3b). This patient did not experience tumor regression after 9 weeks of systemic treatment (TRG 5) and eventually died from recurrent disease 35 months after the initial diagnosis (Supplementary Data 1). Three of the 19 patients identified had ctDNA levels below the limit of detection at baseline, but presented with detectable ctDNA at the preoperative timepoint, suggesting that they did not respond to the preoperative treatment (Supplementary Data 8). Not surprisingly, all of these patients had disease recurrence after surgery and two of the three died from disease (Supplementary Data 1).

After preoperative chemotherapy, we identified seven responders, of whom three achieved complete pathological response (TRG 1) and four achieved a major pathological response, exhibiting fibrotic surgical specimens with scattered tumor cells (TRG 2). All seven responders (100%) had no ctDNA detected at the preoperative timepoint (Fig. 3c, d). Of the 19 patients who showed a measurable but not complete or major pathologic response (TRG 3–4), 13 of these (68%) were ctDNA negative and 10 out of 17 patients without pathologic changes (TRG 5) had detectable ctDNA (59%). Overall, ctDNA analyses were consistent with pathologic response assessment in 30 of the 43 (70%) of patients (*p* = 0.03, Fisher’s exact test) (Fig. 3c, d; Supplementary Fig. 8a).

As expected, recurrence was associated with lower degrees of pathological response (*p* *=* 0.03, Fisher’s exact test) (Fig. 3e), at least one involved lymph node (*p* *=* 0.002, Fisher’s exact test) (Fig. 3f), and detectable ctDNA at the preoperative timepoint (*p* = 0.02, Fisher’s exact test) (Fig. 3g). TRG score (TRG 1–2 vs. TRG 3–5) and pathological lymph node status (ypN0 vs. ypN+) were strongly associated with survival outcomes (Supplementary Fig. 8b, c). It is noteworthy that detection of mutations in cfDNA without a WBC sequence filter at the preoperative timepoint did not predict risk of recurrence (Fig. 3h) or death (Supplementary Fig. 9a). However, when we applied the WBC-guided hematopoietic filter, we observed that ctDNA detected at the preoperative timepoint was associated with a significantly higher risk of recurrence and shorter median event-free survival (18.4 months vs. median not reached) (log-rank *p* = 0.012; hazard ratio (HR) = 3.0; 95% confidence interval (CI) = 1.3–6.9) (Fig. 3i) as well as higher risk of death and shorter median overall survival (28.7 months vs. median not reached) (log-rank *p* = 0.03; HR = 2.7; 95% CI = 1.1–6.7) (Supplementary Fig. 9b).

### MRD predicts survival outcome after surgery

**Fig. 4:**
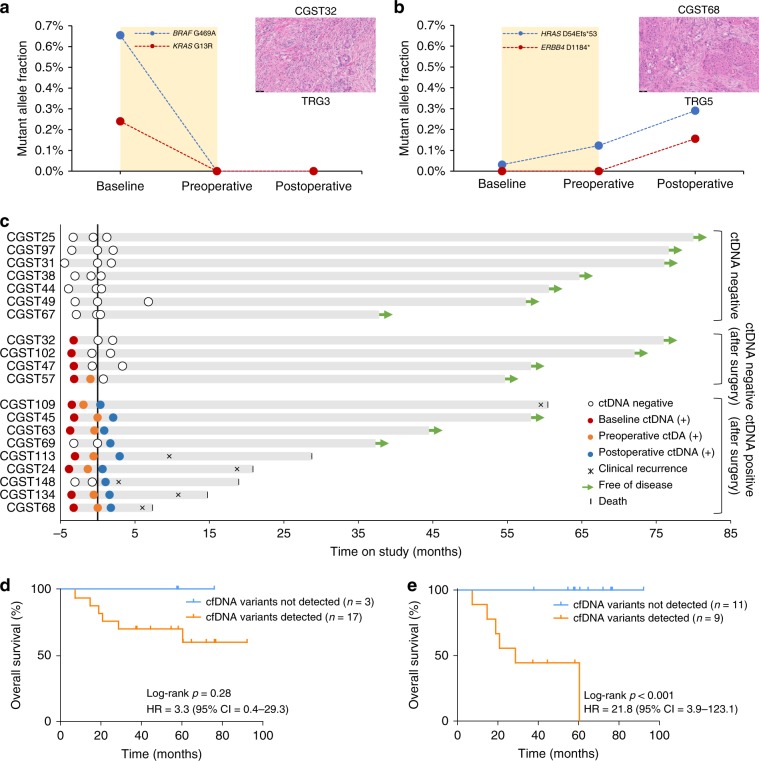
Assessment of ctDNA as a minimal residual disease biomarker in resectable gastric cancer. Levels of ctDNA variants at baseline, preoperative, and postoperative timepoints in a molecular responder (CGST32) (**a**) and in a non-responder (CGST68) (**b**). Variant MAFs in the molecular responder show elimination of ctDNA, while ctDNA levels continue to rise in the molecular non-responder. A representative H&E image (×20 magnification) depicting Mandard’s tumor regression grade is shown for each case on the right. Longitudinal representation of ctDNA results from 20 patients with a postoperative timepoint available. Black vertical line represents the time of surgery. Green arrows depict patients with no evidence of disease at last follow-up (**c**). Kaplan–Meier estimates for overall survival of patients with detected vs. not detected variants at the postoperative timepoint using all cfDNA sequence changes (log-rank *p* *=* 0.28; HR = 3.3; 95% CI = 0.4–29) (**d**) or using only ctDNA alterations identified from the WBC-filtered approach (log-rank *p* = 0.001; HR = 21.8; 95% CI = 3.9–123.1) (**e**).

We used the WBC-filtering approach to evaluate MRD after surgery from all 20 patients with blood samples available from a postoperative timepoint. Blood samples were collected at a median time of 6.5 weeks after surgery (Supplementary Data 1). We observed complete elimination of tumor-specific mutations in cfDNA at the postoperative timepoint for four patients with major tumor responses (TRG 1 and TRG 2), including in patient CGST32, who exhibited baseline mutant allele fraction concentrations of 0.65% and 0.24% for *BRAF* G469A and *KRAS* G13R, respectively (Fig. 4a). The two hotspot ctDNA mutations in this patient were not detected at either the pre- or postoperative timepoint, in agreement with the surgical specimen assessment that showed major tumor regression (TRG 2) (Fig. 4a; Supplementary Data 1 and 8). In contrast, we detected postoperative tumor-specific mutations in 9 out of 16 patients with minor or no pathologic tumor responses (TRG 3–5), including in patient CGST68, who presented with mutant allele fraction of 0.03% for *HRAS* D54Efs*53 frameshift mutation at the baseline timepoint. This patient exhibited progressive increases in mutant allele fractions of *HRAS* at preoperative and postoperative timepoints, followed by the emergence of *ERBB4* D1184*, detected at 0.16% mutant allele fraction after surgery (Fig. 4b; Supplementary Data 1 and 8).

After a median follow-up of 42 months, we observed that all 11 patients without detectable tumor-specific mutations at the postoperative timepoint were alive and free of recurrence (Fig. 4c; Supplementary Data 1). On the other hand, six out of nine patients with detectable tumor-specific mutations at the postoperative timepoint developed disease recurrence and died from metastatic disease (Fig. 4c; Supplementary Data 1). Detection of mutations in cfDNA without a WBC filter after surgery did not predict recurrence (Supplementary Fig. 10a) or death (Fig. 4d). In contrast, with the WBC-guided hematopoietic filter, we observed a significant shorter median event-free survival and a 21.8 times higher risk of disease recurrence for patients with detectable tumor-specific mutations after surgery (18.7 months vs. median not reached; log-rank *p* < 0.001; HR = 21.8; 95% CI = 3.9–123.1) (Supplementary Fig. 10b) as well as a significantly shorter median overall survival (28.7 vs. median not reached; log-rank *p* < 0.001; HR = 21.8; 95% CI = 3.9–123.1) (Fig. 4e). Disease recurrence using ctDNA analyses was determined at 1.3 months, while clinical detection occurred at 10.2 months, resulting in ctDNA median lead time to relapse of 8.9 months (Supplementary Fig. 10c).

## Discussion

High mortality rates associated with gastric cancer reflect the prevalence of advanced disease at presentation, when treatment options are limited^4^. Despite the value of multimodal curative treatment approaches, a significant fraction of patients will eventually perish as a consequence of locoregional relapse, peritoneal recurrence, or distant metastases^2,3^. Current methods to estimate the risk of disease recurrence after surgery mostly rely on the assessment of pathological staging and microscopic residual disease score systems^6–8^. However, there are several limitations with these approaches, especially with tumor regression grading scales, which make their implementation difficult in daily clinical practice^8^. Furthermore, the poor sensitivity of currently available imaging methods and blood protein biomarkers to detect remaining disease after curative surgery has provided an opportunity for ctDNA analyses for MRD assessment in gastric cancer. There is an urgent clinical need to select patients who need adjuvant treatment because of the presence of MRD. Here, we have developed a tissue-independent sequencing approach using ultrasensitive sequencing of matched cfDNA and WBCs to detect tumor-specific mutations in cfDNA after completion of preoperative chemotherapy as well as after surgery in patients with resectable gastric cancer.

In this study, we investigated the value of parallel deep sequencing of cfDNA and WBCs to detect cfDNA alterations associated with clonal hematopoiesis and used this approach to longitudinally infer bona fide tumor-specific alterations independent of matched tumor analyses. Although ctDNA analyses for detection of MRD have been utilized for a variety of solid tumors^13,14,23,27,32,33,47,48^, these efforts have used a tumor-guided approach to identify potential alterations. An advantage of our approach is the identification of ctDNA without requiring tumor tissue, which may be unobtainable or available to a limited extent and where sequencing analyses may be hampered by intra-tumoral heterogeneity.

As the majority of cfDNA typically arises from hematopoietic cells^49^, a major challenge for the development of MRD assays using noninvasive liquid biopsies is distinguishing tumor-specific mutations from background changes associated with biological variation. WBC-derived alterations that arise as a consequence of clonal hematopoiesis may confound liquid biopsy analyses that are based on characterization of cfDNA as these may occur in common cancer driver genes^38–41^. As we have shown here, cfDNA analyses without parallel analyses of WBC DNA would have been unable to appropriately identify patients that benefit from perioperative treatment in terms of event-free and overall survival.

However, there are some limitations when using these approaches to noninvasively detect gastric cancer, especially in patients with localized disease. In this study, although we analyzed a relatively small number of patients with available plasma samples, the extensive follow-up time of the CRITICS trial allowed for accurate determination of clinical recurrences. Despite the fact that the panel we utilized was not developed for gastric cancer we detected the majority of patients at the baseline timepoint. Of the patients not detected at baseline, 61% had diffuse subtype, highlighting potential differences in DNA shedding associated with this histological feature. Some patients did not recur despite positive ctDNA results after surgery, but this observation is consistent with the fact that a fraction of patients receiving adjuvant therapy would be expected to be cured of disease^50,51^ and we did not assess ctDNA levels after adjuvant therapy. Future studies will address the value of post-adjuvant ctDNA status as a surrogate marker for adjuvant treatment efficacy. Tumor-guided personalized approaches may improve the accuracy of such methods^14,32^, but have the disadvantage of requiring tumor tissue and are challenging to implement as widely available standardized tests. Although our approach can be improved in the future with targeted panels specifically designed for gastric cancer and incorporating fragmentation analyses of cfDNA^19^, our efforts provide a proof of principle of the utility of ctDNA analyses in patients receiving perioperative treatment and deserves further validation in randomized interventional trials.

## Methods

### Experimental study design

The current study is a planned exploratory analysis of the predictive value of cfDNA assessment in 50 randomly selected patients from the CRITICS study (NCT00407186) who had plasma samples available and suitable for genomic analyses from at least two timepoints (Fig. 1a; Supplementary Fig. 1; Supplementary Data 1). The CRITICS study is an investigator-initiated, open-label, multi-center, phase III randomized controlled trial of perioperative chemotherapy (chemotherapy group) vs. preoperative chemotherapy with postoperative chemoradiotherapy (chemoradiotherapy group) in patients with resectable gastric cancer^42^. A total of 788 patients from 56 hospitals in the Netherlands, Sweden, and Denmark were randomized upfront to receive three preoperative 21-day cycles of intravenous epirubicin, cisplatin or oxaliplatin, and oral capecitabine followed by three postoperative cycles of intravenous epirubicin, cisplatin or oxaliplatin, and oral capecitabine (chemotherapy group) or to receive the same preoperative regimen followed by postoperative radiotherapy combined with daily capecitabine and weekly cisplatin (chemoradiotherapy group) (Fig. 1a; Supplementary Fig. 1; Supplementary Data 1). Baseline blood samples at the time of trial enrollment were used for both cfDNA and WBC targeted deep sequencing (30,000×), followed by independent variant calling and further tumor-specific mutation detection using the WBC-filtering approach (Fig. 1a). Tumor-specific mutations from the consecutive timepoints were identified using the WBC sequencing data from the same patient at baseline.

### Patients and characteristics

Patients were eligible for the study if they had histologically proven gastric adenocarcinoma (as defined by the American Joint Committee on Cancer, 6th edition), stage IB–IVA^52^, as assessed by esophagogastroduodenoscopy and CT of the chest, abdomen, and pelvis. Patients with tumors of the gastroesophageal junction were permitted to enroll when the bulk of the tumor was predominantly located in the stomach and could therefore consist of Siewert types II (true gastroesophageal junction) and III (subcardial stomach) tumors. Patients with Siewert type I (distal esophagus) tumors were not eligible. An exploratory laparoscopy was indicated when the preoperative CT scan suggested peritoneal carcinomatosis. Patient enrollment and genomic studies were conducted in accordance with the Declaration of Helsinki. The study was approved by the medical ethical committee of the Netherlands Cancer Institute and by the review boards of all participating centers. All patients provided oral and written informed consent for sample acquisition for research purposes.

### Pathological assessment of response, MMR, and EBV status

Pathology slides from the resection specimen from each patient were collected and centrally reviewed by NCTvG to confirm histologic subtypes according to the Lauren’s classification criteria^53^. Histopathological regression was determined by NCTvG according to Mandard’s TRG system: (i) TRG 1, no residual tumor left (pathological complete response); (ii) TRG 2, scattered tumor cells left; (iii) TRG 3, fibrosis outgrows tumor; (iv) TRG4, tumor outgrows fibrosis; and (v) TRG 5, no histological signs of regression (Supplementary Data 1). For detection of EBV, the tumor areas were demarcated on H&E slides of the resection specimens. In case of sufficient amount of tumor tissue, three cores per tumor were taken for construction of a tissue microarray (TMA). TMA sections were cut and used for Epstein–Barr virus-encoded RNA in situ hybridization (EBER-ISH). In case little or no tumor was left in the resection specimen due to chemotherapy-induced pathological (near) complete response, EBER-ISH was performed on the diagnostic biopsy specimen. EBER-ISH was performed using the U INFORM iViEW Blue ISH (v1.02.0023) and the INFORM EBER probe on the Benchmark Ultra IHC/ISH staining module (Roche Diagnostics, the Netherlands) according to the manufacturer’s protocol (Supplementary Data 1).

Formalin-fixed paraffin-embedded tissue blocks from the diagnostic biopsy specimen were used for MSI analysis. The tumor area was demarcated on an H&E slide. DNA was isolated from the demarcated tumor area. MSI analysis was performed using the MSI Analysis System (MSI Multiplex System, version 1.2, Promega) consisting of five nearly monomorphic mononucleotide markers (*BAT-25*, *BAT-26*, *NR-21*, *NR-24*, *MONO-27*) according to the manufacturer’s instructions. PCR products were separated by capillary electrophoresis using an ABI 3500 Genetic Analyzer (Applied Biosystems, Foster City, CA, USA), and analyzed using the GeneMapper software (Applied Biosystems, Foster City, CA, USA). An internal lane size standard was added to the PCR samples for accurate sizing of alleles and to adjust for run-to run variations. When all markers were stable, the tumor was interpreted as microsatellite stable (MSS). The tumor was interpreted as MSI-low (MSI-L) if one marker was unstable and MSI-high (MSI-H) if two or more markers showed instability. MSI-L tumors were included in the MSS category (Supplementary Data 1).

### Next-generation sequencing of cfDNA and DNA from WBCs

Whole blood was collected in K_2_EDTA tubes, sent to the central pathology lab at VUmc, Amsterdam, and plasma and cellular components were separated by centrifugation at 1300 r.p.m. for 5 min in 1.5 ml microcentrifuge tubes at 4 °C and stored at −20 °C until the time of DNA extraction. Based on our previous analyses of ctDNA stability^54^, we only included samples in this study that were processed within 24 h after collection. cfDNA was isolated from plasma using the Qiagen Circulating Nucleic Acids Kit (Qiagen GmbH) and eluted in LoBind tubes (Eppendorf AG). High-molecular weight DNA from WBCs was extracted using the Qiagen DNA Blood Mini Kit (Qiagen GmbH), followed by shearing using a focused-ultrasonicator (Covaris). Concentration and quality of cfDNA was assessed using the Bioanalyzer 2100 (Agilent Technologies). cfDNA samples with high-molecular weight DNA were excluded from the study.

Next-generation sequencing libraries from cfDNA and sheared high-molecular weight DNA from WBCs were prepared from 8.4 to 250 ng (Supplementary Data 2). Genomic libraries were prepared as previously described^18^. Briefly, the NEBNext DNA Library Prep Kit for Illumina [New England Biolabs (NEB)] was used with four main modifications to the manufacturer’s guidelines: (i) the library purification steps utilized the on-bead Ampure XP approach, (ii) reagent volumes were adjusted accordingly to accommodate the on-bead strategy, (iii) a pool of eight unique Illumina dual index adapters with 8 bp barcodes were used in the ligation reaction, and (iv) cfDNA libraries were amplified with HotStart Phusion Polymerase. Genomic library preparation was performed as previously described^18^. Concentration and quality of cfDNA genomic libraries were assessed using the Bioanalyzer 2100 (Agilent Technologies).

Targeted capture was performed using the Agilent SureSelect reagents and a custom set of hybridization probes targeting 58 genes (Supplementary Data 3) as per the manufacturer’s guidelines. The captured library was amplified with HotStart Phusion Polymerase (NEB). The concentration and quality of captured cfDNA libraries was assessed on the Bioanalyzer (Agilent Technologies). Libraries were sequenced using 100-bp paired-end runs on the Illumina HiSeq 2500 (Illumina).

### Identification of candidate somatic mutations in cfDNA

Primary processing of next-generation sequence data for analyses of sequence alterations in cfDNA and WBC samples were performed as previously described^18^. Briefly, Illumina CASAVA (Consensus Assessment of Sequence and Variation) software (version 1.8) was used for demultiplexing and masking of dual index adapter sequences. Sequence reads were aligned against the human reference genome (hg19) using NovoAlign with additional realignment of select regions using the Needleman–Wunsch method^55^.

Candidate tumor-specific mutations in cfDNA, consisting of point mutations, small insertions, and deletions were identified using VariantDx^55^ (Personal Genome Diagnostics) across the targeted regions of interest as previously described^18^. Briefly, an alteration was considered a candidate somatic mutation only when: (i) three distinct paired reads contained the mutation in the cfDNA and the number of distinct paired reads containing a particular mutation in the plasma was at least 0.05% of the total distinct read pairs; or (ii) one distinct paired read contained the mutation in the cfDNA and the mutation had also been detected in at least one additional timepoint at the level specified in (i); (iii) the mismatched base or small indel was not identified in matched WBC sequencing data of samples collected at baseline at the level of one distinct read (Supplementary Data 8); (iv) the mismatched base or small indel was not present in a custom database of common germline variants derived from dbSNP; (v) the altered base did not arise from misplaced genome alignments including paralogous sequences; and (vi) the mutation fell within a protein coding region and was classified as a missense, nonsense, frameshift, or splice site alteration. Candidate alterations were defined as somatic hotspots if the nucleotide change and amino acid change were identical to an alteration observed in ≥20 cancer cases reported in the COSMIC database.

### Probability model of tumor-specific mutations detected in cfDNA

For mutations identified by cfDNA sequencing but not identified by WBC sequencing, we computed the probability that the mutation was tumor derived relative to the probability that the mutation was hematopoietic. The sampling distribution of the observed number of reads with an altered mutation in cfDNA and WBC sequencing is a binomial parameterized by the total coverage at that mutation and unknown probability *θ*. Under the tumor-derived model, *θ*_WBC_ is zero and only *θ*_CFDNA_ is unknown. For the hematopoietic model, we assume that *θ*_WBC_ and *θ*_CFDNA_ are the same. As a prior for *θ*_CFDNA_, we used a *β* distribution with shape parameters 2.4 and 340 that loosely centers most of the mass on the observed mutation allele frequencies in samples for which mutations were identified in both cfDNA and WBC sequencing. This prior is equivalent to a sample with 2.4 altered reads per 340 distinct molecules. Sampling a large number of *θ*s from the prior, we computed the probability of the observed data for each simulated *θ*. The ergodic average of these probabilities approximates the likelihood of the observed data conditional on the model, but unconditional on *θ*. Assuming a prior odds of 1, the posterior odds (POs) was the same as the Bayes factor and we obtained the probability that the mutation was tumor derived by PO/(1 + PO). We performed this analysis for each mutation that was identified only by cfDNA sequencing.

### Statistical analyses

Statistical significance was determined using a variety of methods. Wilcoxon’s rank-sum test or Kruskal–Wallis test were performed for two- and three-way comparisons of continuous variables. Independence of categorical variables was evaluated by Fisher’s exact test and *χ*^2^ tests. Visualizations of groups of mutations were carried out in R using the package *maftools*^56^. Pearson’s correlation coefficients were determined for the association between WBC variants and their correspondent alterations identified in cfDNA, as well as for the association between the number of WBC variants and age. Differences in survival between two groups were evaluated using log-rank tests and implemented in R using the survival package. All hypothesis tests were two sided.

### Reporting summary

Further information on research design is available in the Nature Research Reporting Summary linked to this article.

## Supplementary information

Supplementary Information

Description of Additional Supplementary Files

Supplementary Data 1

Supplementary Data 2

Supplementary Data 3

Supplementary Data 4

Supplementary Data 5

Supplementary Data 6

Supplementary Data 7

Supplementary Data 8

Supplementary Data 9

Supplementary Data 10

Reporting Summary

## Data Availability

The sequencing data from cfDNA and WBC samples have been deposited at the European Genome Phenome Archive under the accession code EGAS00001004114. All the other data supporting the findings of this study are available within the article and its supplementary information files and from the corresponding author upon reasonable request. A reporting summary for this article is available as a Supplementary Information file.
