# Supplementary material for: White blood cell and cell-free DNA analyses for detection of residual disease in gastric cancer
Source: Nat Commun. 2020 Jan 27;11:525. doi: 10.1038/s41467-020-14310-3 (PMC6985115; doi:10.1038/s41467-020-14310-3)
Supplement: Supplementary file 1 — Supplementary Information [file 41467_2020_14310_MOESM1_ESM.pdf]

Supplementary Information for

Matched white blood cell and cell-free DNA analyses for detection of minimal residual disease in patients with gastric cancer

Alessandro Leal[1*], Nicole C. T. van Grieken[2*], Doreen N. Palsgrove[1], Jillian Phallen[1], Jamie E. Medina[1], Carolyn Hruban[1], Mark A. M. Broeckaert[2], Valsamo Anagnostou[1], Vilmos Adleff[1], Daniel C. Bruhm[1], Jenna V. Canzoniero[3], Jacob Fiksel[1], Marianne Nordsmark[4], Fabienne A. R. M. Warmerdam[5], Henk M. W. Verheul[6], Dick Johan van Spronsen[7], Laurens V. Beerepoot[8], Maud M. Geenen[9], Johanneke E. A. Portielje[10†], Edwin P. M. Jansen[11], Johanna van Sandick[12], Elma Meershoek-Klein Kranenbarg[13], Hanneke W. M. van Laarhoven[14], Donald L. van der Peet[15], Cornelis J. H. van de Velde[13], Marcel Verheij[11], Remond Fijneman[16], Robert B. Scharpf[1], Gerrit A. Meijer[16], Annemieke Cats[17], Victor E. Velculescu[1††]

[1]The Sidney Kimmel Comprehensive Cancer Center, Johns Hopkins University School of Medicine, Baltimore, MD 21287, USA

[2]Department of Pathology, Cancer Center Amsterdam, Amsterdam UMC, Vrije Universiteit, Amsterdam, Netherlands

[3]Department of Medicine, Johns Hopkins University School of Medicine, Baltimore, MD 21287, USA

[4]Department of Oncology, Aarhus University Hospital, Aarhus, Denmark

[5]Department of Medical Oncology, Zuyderland Medical Centre, Sittard-Geleen/Heerlen, Netherlands

[6]Department of Medical Oncology, Cancer Center Amsterdam, Amsterdam UMC, Vrije Universiteit, Amsterdam, Netherlands

[7]Department of Hematology, Radboud University Nijmegen Medical Centre, Nijmegen, Netherlands

[8]Department of Internal Medicine, St Elisabeth-Tweesteden Ziekenhuis, Tilburg, Netherlands

[9]Department of Internal Medicine, Onze Lieve Vrouwe Gasthuis, Amsterdam, Netherlands

[10]Department of Internal Medicine, HAGA hospital, The Hague, Netherlands

[11]Department of Radiation Oncology, Netherlands Cancer Institute, Amsterdam, Netherlands

[12]Department of Surgery, Netherlands Cancer Institute, Amsterdam, Netherlands

[13]Department of Surgery, Leiden University Medical Center, Leiden, Netherlands

[14]Department of Medical Oncology, Cancer Center Amsterdam, Amsterdam UMC, Amsterdam, Netherlands

[15]Department of Surgery, Cancer Center Amsterdam, Amsterdam UMC, Vrije Universiteit, Amsterdam, Netherlands

[16]Department of Pathology, Diagnostic Oncology, Netherlands Cancer Institute, Amsterdam, Netherlands

[17]Department of Gastrointestinal Oncology, Netherlands Cancer Institute, Amsterdam, Netherlands

*These authors contributed equally to this effort

†Current affiliation: Department of Internal Medicine, Leiden University Medical Center, Leiden, The Netherlands

††Corresponding author: Victor Velculescu, M.D., Ph.D.: velculescu@jhmi.edu

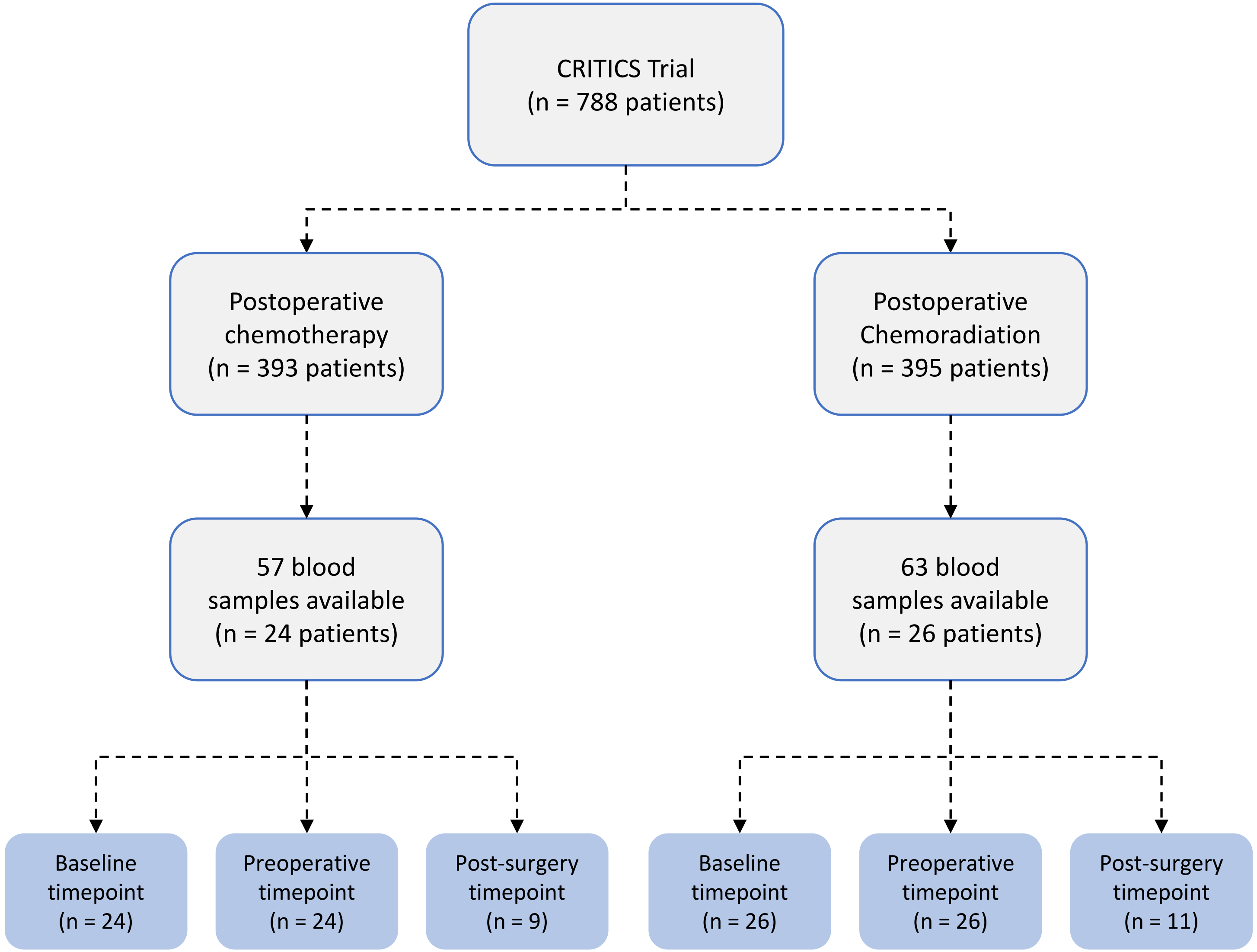

Supplementary Figure 1. Consort diagram of patients enrolled in the CRITICS trial cohort. Patients with operable stage IB-IVA gastric cancer (n = 788) were initially treated with three cycles of preoperative chemotherapy with epirubicin, cisplatin or oxaliplatin, and oral capecitabine followed by surgery. They were randomly assigned to receive postoperative treatment with the same chemotherapy regimen (n = 393) or postoperative radiation with cisplatin and oral capecitabine (n = 395). Plasma samples used for ctDNA analyses were provided by study centers in The Netherlands.

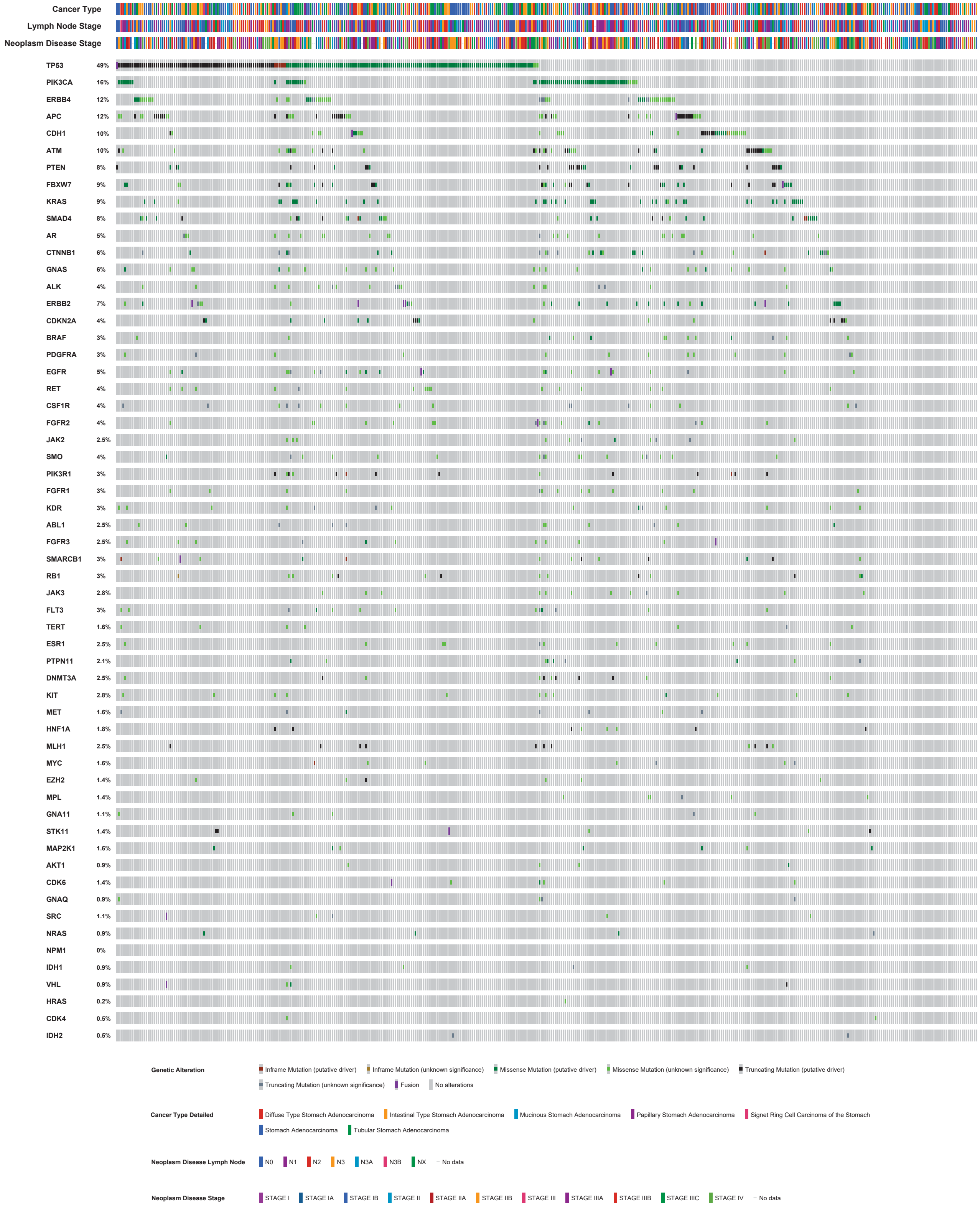

Supplementary Figure 2. Theoretical sensitivity of detection of ultrasensitive NGS approach in gastric cancer. Analyses of TCGA Pan-Cancer Atlas gastric adenocarcinoma cohort showed a theoretical sensitivity of 88 % for the 58-gene panel (81 Kb) used in this study, with 385 out of 436 cases potentially identified using this approach.

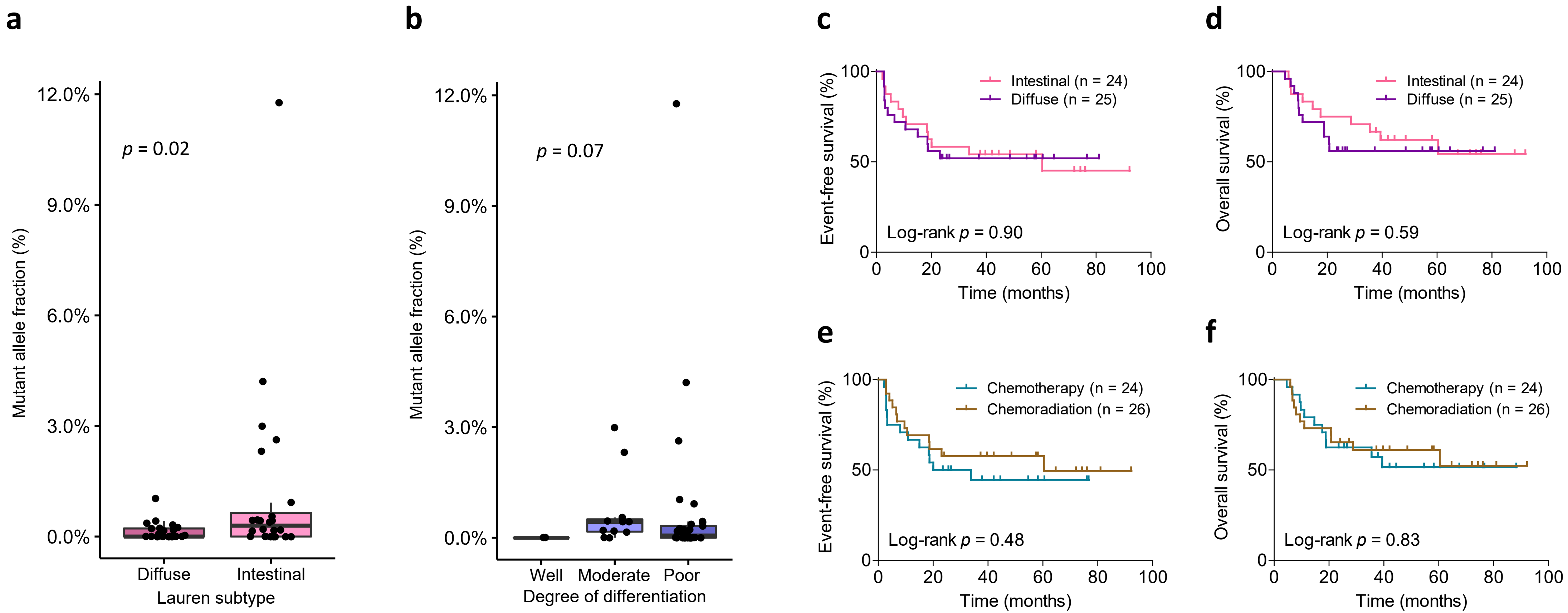

Supplementary Figure 3. Pathological features and ctDNA levels of gastric cancers analyzed. Mutant allele fractions of ctDNA at base line in patients with diffuse and intestinal subtypes (Wilcoxon rank sum test, $p = 0.02$) (a). Mutant allele fractions of ctDNA at baseline in patients with well, moderately, and poorly differentiated tumors (Kruskal -Wallis test, p= 0.07) (b). Kaplan-Meier estimates for event-free survival [Log-rank $p = 0.90$; HR = 1.0 (95% CI = 0.4-2.1)] (c) and overall survival [Log-rank $p = 0.59$; HR = 0.8 [(95% CI = 0.3-1.9)] (d) of patients with intestinal and diffuse subtypes. Kaplan-Meier estimates for event-free survival [Log-rank $p = 0.48$; HR = 1.3 (95% CI = 0.6-3. 0)] (e) and overall survival [Log-rank $p = 0.83$; HR = 1.1 (95% CI = 0.5-2.5)] (f) of patients treated with adjuvant chemotherapy or chemoradiotherapy. Data in (e) and (f) represent a subset of the results previously published by Cats A., et al.[1]

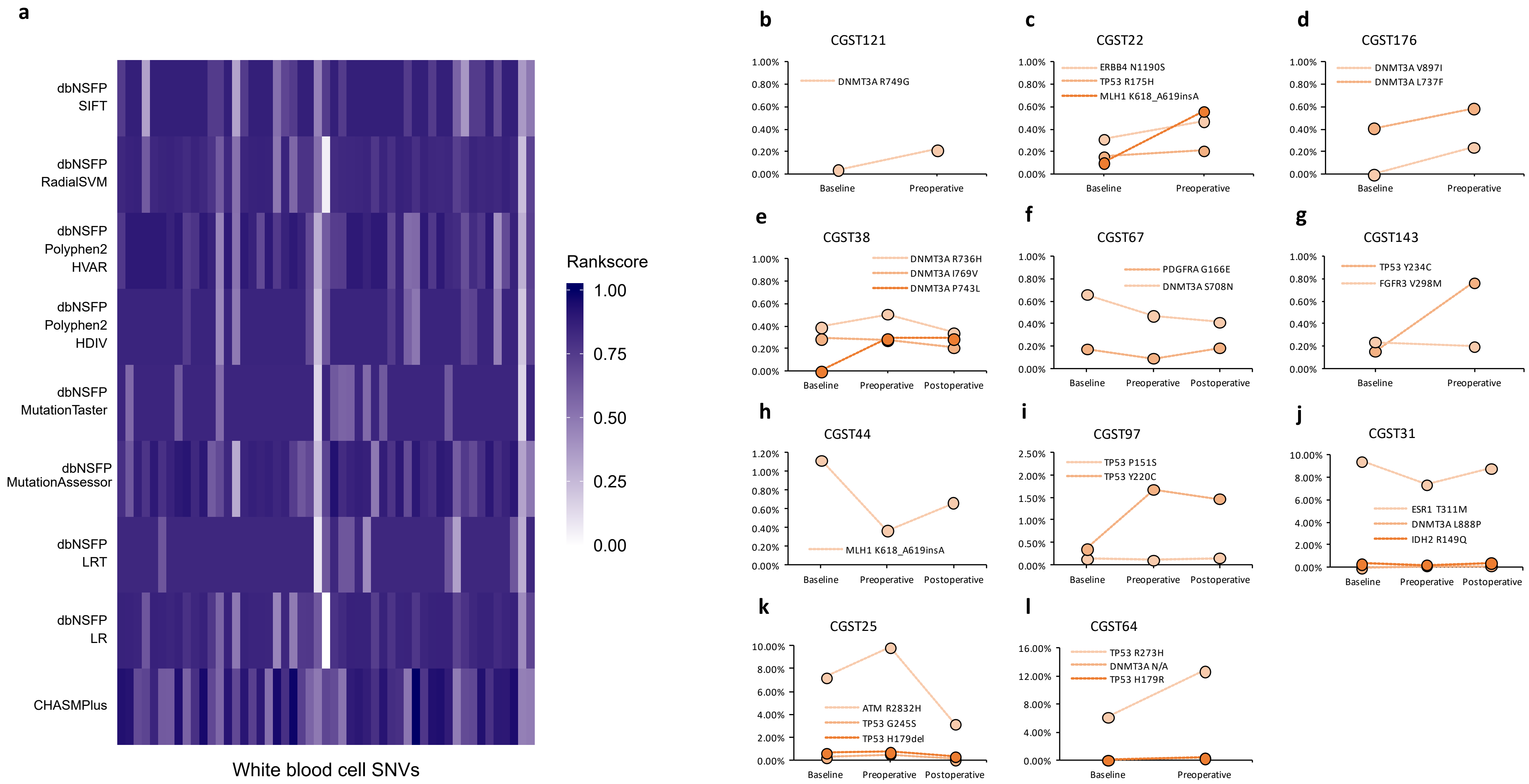

Supplementary Figure 4: Variants detected in white blood cells in patients with gastric cancer. Functional prediction analyses of non-synonymous SNVs detected in white blood cells are indicated for 51 missense variants with a normalized rankscore that represents the variants' pathogenicity (a) (Extended Data Table 10). Fluctuations of the levels of white blood cell variants in patients without tumor-specific mutations detected (b-l).

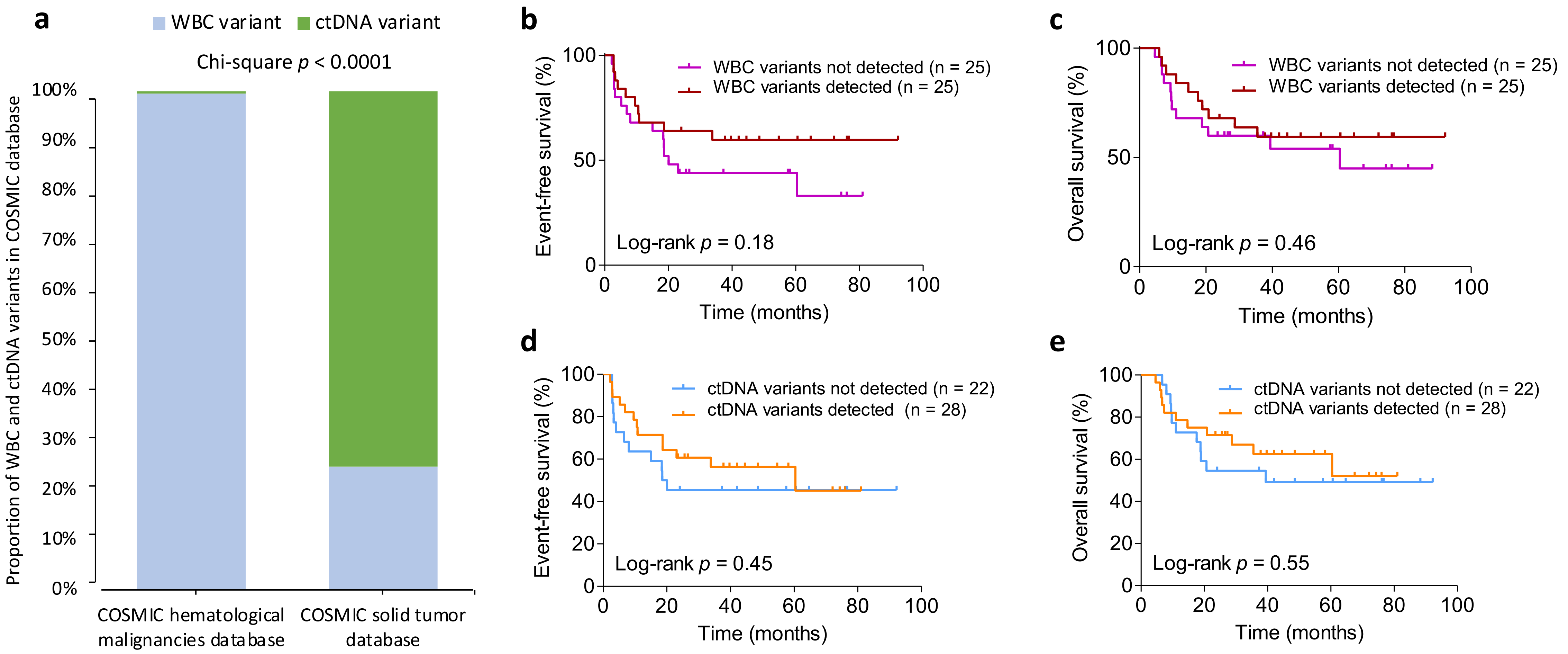

Supplementary Figure 5. Proportion of WBC and ctDNA variants in COSMIC database and survival outcomes at the baseline timepoint. WBC variants detected by the hematopoietic filter approach were less likely found in the COSMIC solid tumor database when compared to ctDNA variants (Chi-square $p < 0.0001$) (a). Kaplan-Meier estimates for event-free survival [Log-rank $p = 0.18$; HR = 1.7 (95% CI = 0.8-3.8)] (b) and overall survival [Log-rank $p = 0.46$; HR = 1.4 (95% CI = 0.6-3.2)] (c) of patients with or without WBCs variants in cfDNA at the time of study enrollment. Kaplan-Meier estimates for event-free survival [Log-rank $p = 0.45$; HR = 1.4 (95%CI = 0.6-3.0)] (d) and overall survival [Log-rank $p = 0.55$; HR = 1.3 (95% CI = 0.6-3.0)] (e) of patients with or without ctDNA detected at the time of study enrollment.

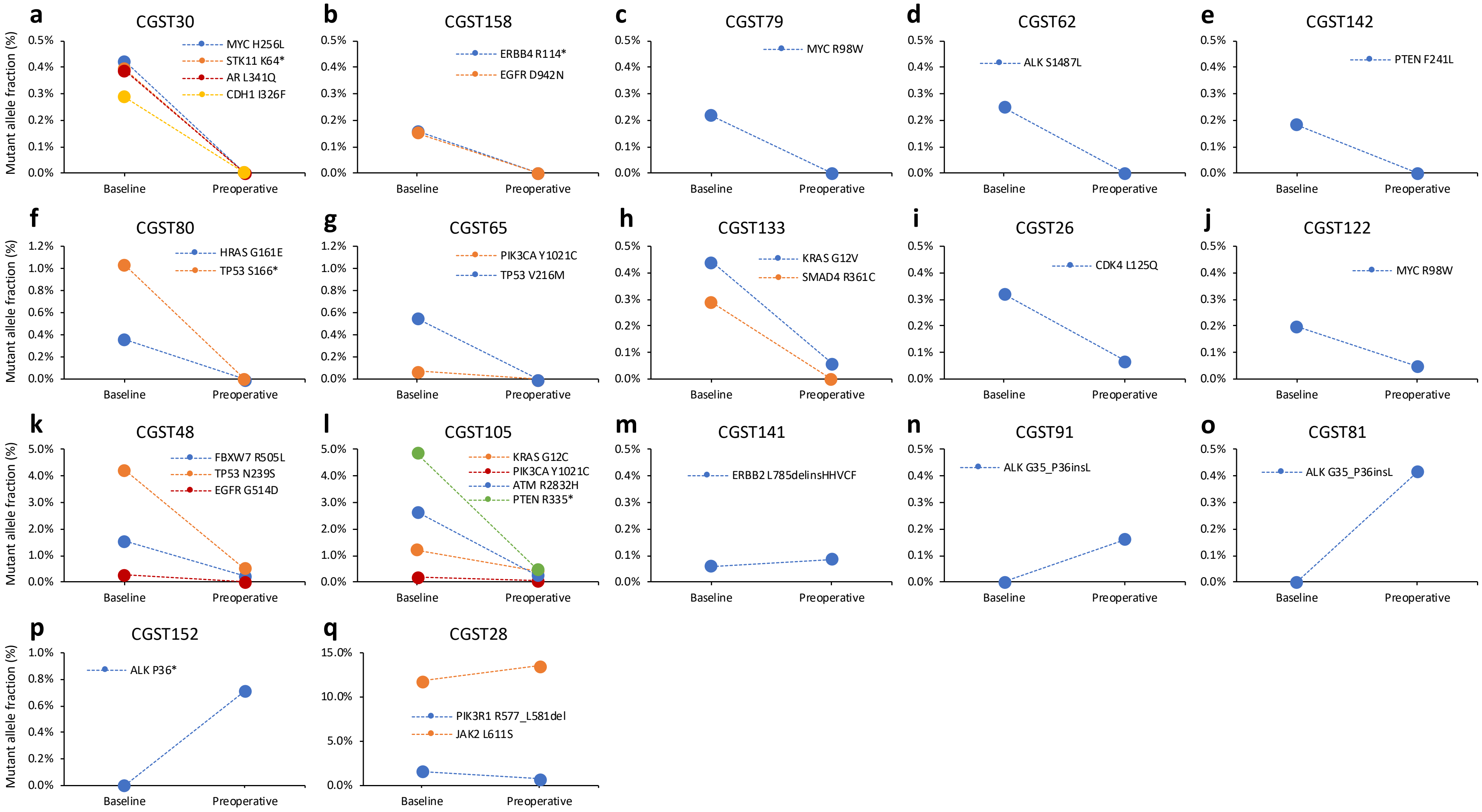

Supplementary Figure 6. Dynamic changes in ctDNA during the preoperative chemotherapy interval. ctDNA alterations in each patient were detected during the preoperative chemotherapy interval after removing WBC variants observed in cfDNA (a-q).

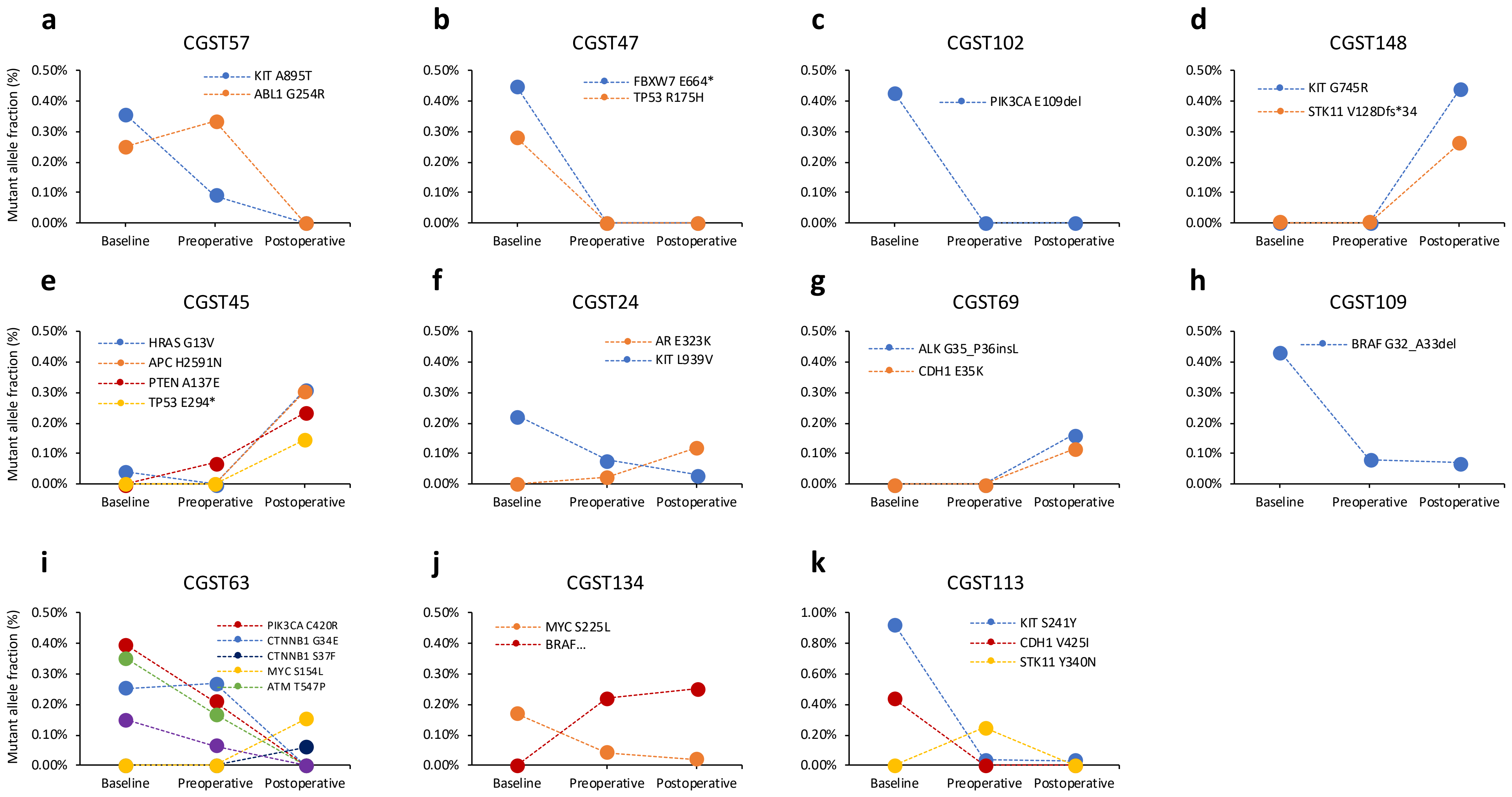

Supplementary Figure 7. Dynamic changes in ctDNA before and after surgery. ctDNA alterations observed for each patient were detected from baseline through postoperative timepoints after removing WBC variants observed in cfDNA (a-k).

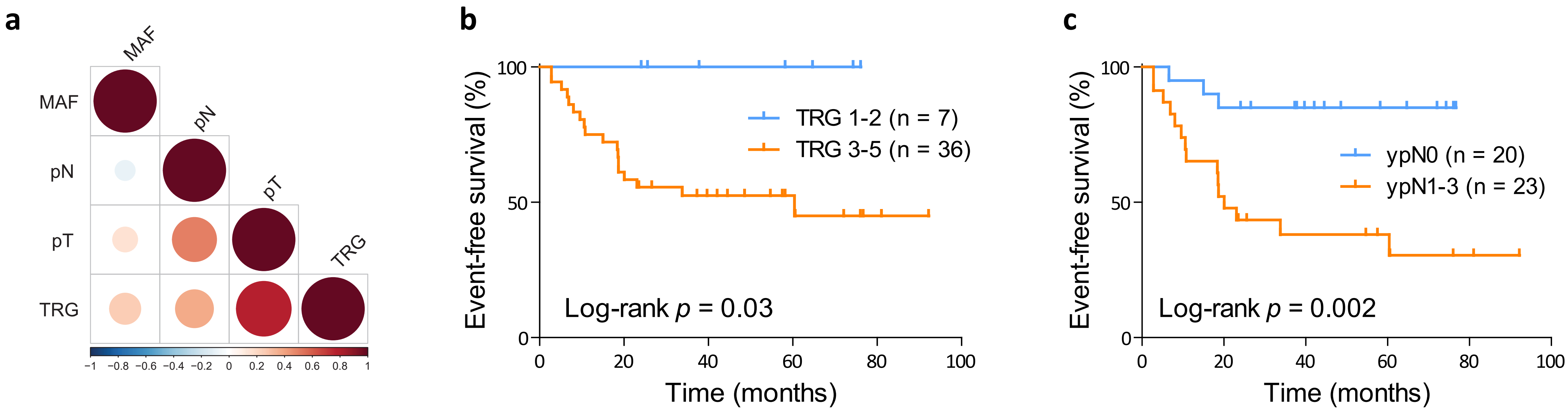

Supplementary Figure 8. Pathological response at the preoperative timepoint and survival outcomes. Heatmap showing the Spearman's rank correlation coefficients between mutant allele fractions at the preoperative timepoint and pathological features after surgery (ypN, pathological lymph node assessment; ypT, pathological tumor assessment; TRG, tumor regression grade) (a). Kaplan-Meier estimates for event-free survival of patients with minor or no pathological response (TRG 3-5) and major pathological responses (TRG 1-2) to preoperative chemotherapy [Log-rank p = 0.03; HR = 3.6 (95% CI = 1.1-11.3)] (b). Kaplan-Meier estimates for event-free survival of patients with (ypN1-3) and without (ypN0) lymph node tumor infiltration [Log-rank p = 0.002; HR = 4.5 (95% CI = 1.8-11.6)] (c).

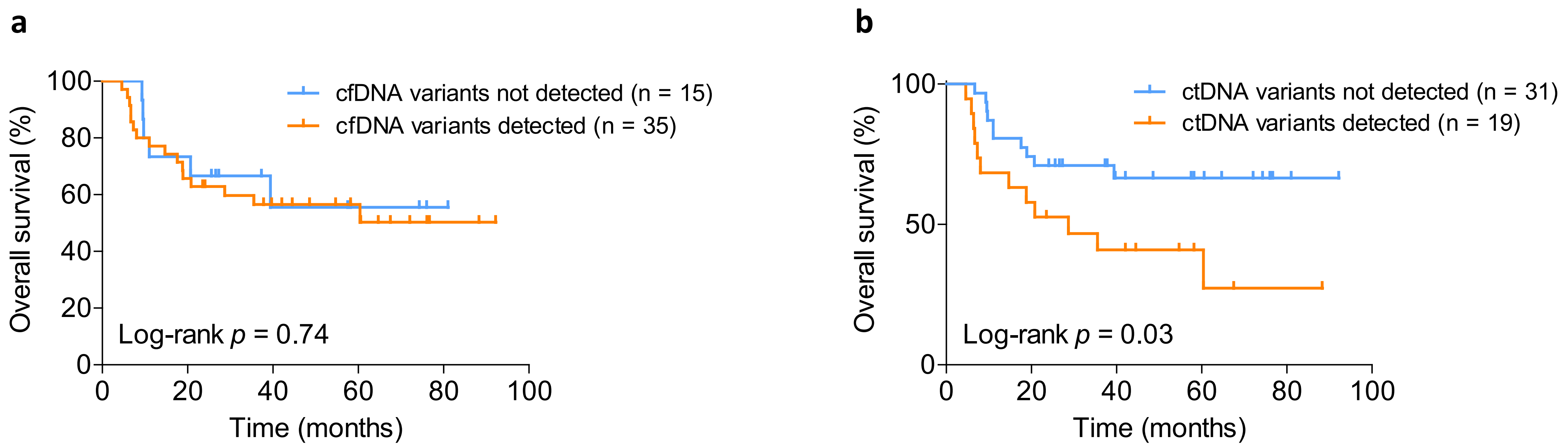

Supplementary Figure 9. Detection of cfDNA and ctDNA variants at the preoperative timepoint and overall survival. Kaplan-Meier estimates for overall survival of patients with detected versus not detected variants at the preoperative timepoint using all cfDNA sequence changes [Log-rank p = 0.74; HR = 0.9 (95% CI = 0.4-2.1)] (a) or using only ctDNA alterations identified from the WBC-filtered approach [Log-rank p = 0.03; HR = 2.7 (95 % CI = 1.1-6.7)] (b).

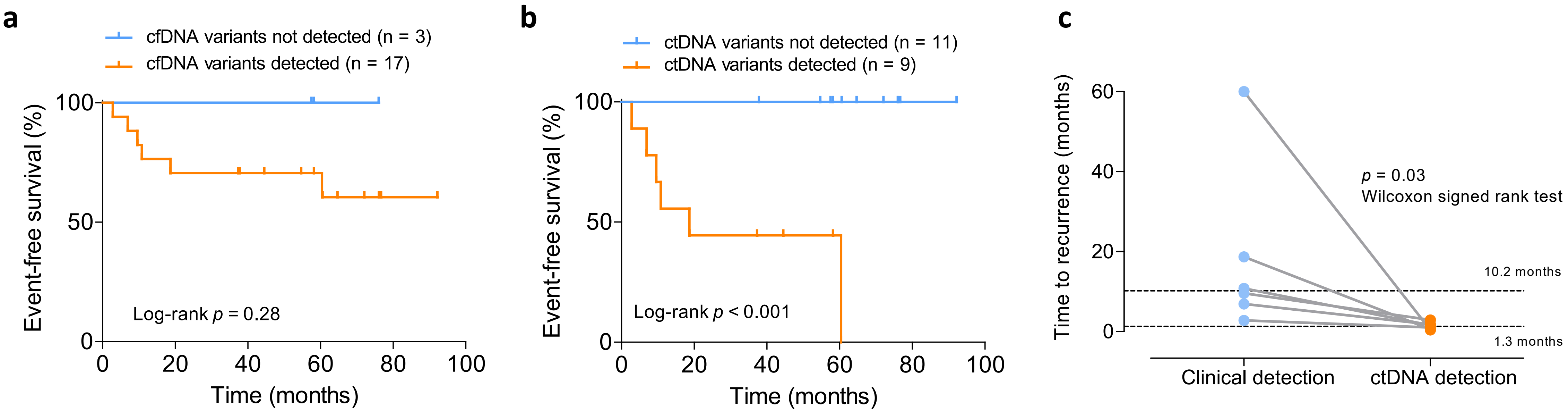

Supplementary Figure 10. Detection of cfDNA and ctDNA variants at the postoperative timepoint and event-free survival. Kaplan-Meier estimates for event-free survival of patients with detected versus not detected variants at the postoperative timepoint using all cfDNA sequence data [Log-rank $p = 0.28$; HR = 3.3 (95% CI = 0.4-29.3)] (a) or using only ctDNA alterations identified from the WBC-filtered approach [Log-rank $p < 0.001$; HR = 21.8 (95% CI = 3.9-123.1)] (b). Time of disease recurrence as determined by clinical and CT imaging assessment (blue) or analyses of ctDNA after surgery (orange) are indicated with median time to recurrence shown in dotted lines ($p = 0.03$, Wilcoxon signed rank test) (c).

Supplementary references

1) Cats, A. et al. Chemotherapy versus chemoradiotherapy after surgery and preoperative chemotherapy for resectable gastric cancer (CRITICS): an international, open-label, randomised phase 3 trial. Lancet Oncol 19, 616-628, doi:10.1016/S1470-2045(18)30132-3 (2018).
