# Supplementary material for: White blood cell and cell-free DNA analyses for detection of residual disease in gastric cancer
Source: Nat Commun. 2020 Jan 27;11:525. doi: 10.1038/s41467-020-14310-3 (PMC6985115; doi:10.1038/s41467-020-14310-3)
Supplement: Supplementary file 2 — Description of Additional Supplementary Files [file 41467_2020_14310_MOESM2_ESM.pdf]

Description of Additional Supplementary Files

File Name: Supplementary Data 1
Description: Clinical characteristics of patients analyzed

File Name: Supplementary Data 2
Description: Serial timepoints analyzed

File Name: Supplementary Data 3
Description: Genes analyzed

File Name: Supplementary Data 4
Description: Summary of genomic analyses of plasma samples

File Name: Supplementary Data 5
Description: Summary of genomic analyses of white blood cell samples

File Name: Supplementary Data 6
Description: Sequence alterations detected in cfDNA

File Name: Supplementary Data 7
Description: Sequence alterations detected in WBCs

File Name: Supplementary Data 8
Description: WBC sequence alterations identified in cfDNA

File Name: Supplementary Data 9
Description: Sequence alterations detected in ctDNA

File Name: Supplementary Data 10
Description: Functional prediction analyses of WBC alterations
