# Supplementary material for: White blood cell and cell-free DNA analyses for detection of residual disease in gastric cancer
Source: Nat Commun. 2020 Jan 27;11:525. doi: 10.1038/s41467-020-14310-3 (PMC6985115; doi:10.1038/s41467-020-14310-3)
Supplement: Supplementary file 13 — Reporting Summary [file 41467_2020_14310_MOESM13_ESM.pdf]

nature research

Corresponding author(s): Victor E. Velculescu

Last updated by author(s): Dec 11, 2019

Reporting Summary

Nature Research wishes to improve the reproducibility of the work that we publish. This form provides structure for consistency and transparency in reporting. For further information on Nature Research policies, see Authors & Referees and the Editorial Policy Checklist .

Statistics

For all statistical analyses, confirm that the following items are present in the figure legend, table legend, main text, or Methods section.

n/a	Confirmed	
☐	☒	The exact sample size (*n*) for each experimental group/condition, given as a discrete number and unit of measurement
☐	☒	A statement on whether measurements were taken from distinct samples or whether the same sample was measured repeatedly
☐	☒	The statistical test(s) used AND whether they are one- or two-sided<br>*Only common tests should be described solely by name; describe more complex techniques in the Methods section.*
☐	☒	A description of all covariates tested
☐	☒	A description of any assumptions or corrections, such as tests of normality and adjustment for multiple comparisons
☐	☒	A full description of the statistical parameters including central tendency (e.g. means) or other basic estimates (e.g. regression coefficient) AND variation (e.g. standard deviation) or associated estimates of uncertainty (e.g. confidence intervals)
☐	☒	For null hypothesis testing, the test statistic (e.g. *F*, *t*, *r*) with confidence intervals, effect sizes, degrees of freedom and *P* value noted<br>*Give P values as exact values whenever suitable.*
☐	☒	For Bayesian analysis, information on the choice of priors and Markov chain Monte Carlo settings
☒	☐	For hierarchical and complex designs, identification of the appropriate level for tests and full reporting of outcomes
☐	☒	Estimates of effect sizes (e.g. Cohen's *d*, Pearson's *r*), indicating how they were calculated

Our web collection on statistics for biologists contains articles on many of the points above.

Software and code

Policy information about availability of computer code

Data collection	Primary processing of targeted NGS data for cfDNA samples was performed using Illumina CASAVA (version 1.8.2) with alignment using NovoAlign (version 3.02.12). Variant calling was performed using VariantDx.
Data analysis	All analyses were performed using R (version 3.4.3). Analyses of groups of mutations were carried out using the package maftools (https://bioconductor.org/packages/devel/bioc/vignettes/maftools/inst/doc/maftools.html). Univariate survival analyses were carried out in R using packages survival and coxphf (https://cran.rproject.org).

For manuscripts utilizing custom algorithms or software that are central to the research but not yet described in published literature, software must be made available to editors/reviewers. We strongly encourage code deposition in a community repository (e.g. GitHub). See the Nature Research guidelines for submitting code & software for further information.

Data

Policy information about availability of data

All manuscripts must include a data availability statement. This statement should provide the following information, where applicable:

- Accession codes, unique identifiers, or web links for publicly available datasets
- A list of figures that have associated raw data
- A description of any restrictions on data availability

The sequencing data from cfDNA and white blood cell samples have been deposited at the European Genome Phenome Archive (EGAS00001004105).

Field-specific reporting

Please select the one below that is the best fit for your research. If you are not sure, read the appropriate sections before making your selection.

Life sciences study design

All studies must disclose on these points even when the disclosure is negative.

Sample size	A total of 120 serial plasma samples from 50 patients would be needed to provide an estimate of the difference of disease recurrence of at least 45% between groups with ctDNA detected (assuming an incidence of recurrence of 75%) and not detected (assuming an incidence of recurrence of 30%) after preoperative chemotherapy in patients with resectable gastric cancer eligible for multi-modal therapy, with a 90% power at an $\alpha = 0.05$.
Data exclusions	Plasma samples that were not collected according the described protocol were not used in the analysis.
Replication	Not applicable
Randomization	Not applicable
Blinding	Not applicable

Reporting for specific materials, systems and methods

We require information from authors about some types of materials, experimental systems and methods used in many studies. Here, indicate whether each material, system or method listed is relevant to your study. If you are not sure if a list item applies to your research, read the appropriate section before selecting a response.

Materials & experimental systems

n/a	Involved in the study	
☒	☐	Antibodies
☒	☐	Eukaryotic cell lines
☒	☐	Palaeontology
☒	☐	Animals and other organisms
☐	☒	Human research participants
☐	☒	Clinical data

Methods

n/a	Involved in the study	
☒	☐	ChIP-seq
☒	☐	Flow cytometry
☒	☐	MRI-based neuroimaging

Human research participants

Policy information about studies involving human research participants

Population characteristics	Patients were eligible for this translational study if they had plasma samples available and suitable for genomic analyses, as well as histologically proven gastric adenocarcinoma, stage IB-IVA, as assessed by esophagogastroduodenoscopy and CT of the chest, abdomen, and pelvis. Patients with tumors of the gastroesophageal junction were permitted to enroll when the bulk of the tumor was predominantly located in the stomach and could therefore consist of Siewert types II (true gastroesophageal junction) and III (subcardial stomach) tumors.
Recruitment	Patients in this study were recruited from centers in the Netherlands (44 hospitals) that participated in the CRITICS trial (NCT00407186; Cats et al., Lancet Oncol, 2018). CRITICS trial is an investigator-initiated, open-label, multi-center, phase III study of perioperative chemotherapy (chemotherapy group) versus preoperative chemotherapy with postoperative chemoradiotherapy (chemoradiotherapy group) in patients with resectable gastric cancer. Patients with blood samples (plasma and white blood cells) available for at least two timepoitns during the course of treatment were included in this study. Potential self-selection bias or other biases were not identified.
Ethics oversight	The study was approved by the medical ethical committee of the Netherlands Cancer Institute and by the review boards of all participating centers. All patients provided oral and written informed consent.

Note that full information on the approval of the study protocol must also be provided in the manuscript.

Clinical data

Policy information about clinical studies

All manuscripts should comply with the ICMJEguidelines for publication of clinical research and a completedCONSORT checklist must be included with all submissions.

Clinical trial registration	ClinicalTrials.gov number: NCT00407186<br>EudraCT number: 2006-004130-32
Study protocol	The full trial protocol can be assessed at Cats et al., Lancet Oncol, 2018 (DOI: https://doi.org/10.1016/S1470-2045(18)30132-3)

Data collection	Period of recruitment and data collection: from Jan 11, 2007 to April 17, 2015.<br><br>A total of 788 patients from 56 hospitals in the Netherlands, Sweden, and Denmark were randomized upfront to receive three preoperative 21-day cycles of intravenous epirubicin, cisplatin or oxaliplatin, and oral capecitabine followed by three postoperative cycles of intravenous epirubicin, cisplatin or oxaliplatin, and oral capecitabine (chemotherapy group) or to receive the same preoperative regimen followed by radiation combined with daily capecitabine and weekly cisplatin. In the actual study, we analyzed 50 patients with blood samples available at multiple timepoints.<br><br>On-site data monitoring was done with source verification for informed consent, inclusion and exclusion criteria, protocol procedures, missing data, and serious adverse events for at least the first five registered patients in all participating centres. Surgical and pathological quality assurance involved central review for type and completeness of resection, including number of lymph nodes retrieved (NvG). Radiotherapy quality assurance consisted of pre-treatment assessment of treatment plans of at least the first three patients included by each radiotherapy institute, and for subsequent patients if deemed necessary by the principle investigators (EPMJ and MV), or requested by the treating radiation oncologist. Target volume delineation manuals and workshops were offered to all participating institutions.
Outcomes	The primary endpoint was overall survival, defined as the time from randomization to death from any cause. Secondary endpoint was event-free survival, defined as time from randomization until disease progression, irresectable disease at surgery, locoregional or peritoneal tumor recurrence, distant metastases, or death from any cause, whichever occurred first.
